# A Novel Correction Methodology to Improve the Performance of a Low-Cost Hyperspectral Portable Snapshot Camera

**DOI:** 10.3390/s23249685

**Published:** 2023-12-07

**Authors:** Andrea Genangeli, Giovanni Avola, Marco Bindi, Claudio Cantini, Francesco Cellini, Ezio Riggi, Beniamino Gioli

**Affiliations:** 1 Department of Agriculture, Food, Environment and Forestry (DAGRI), University of Florence, P. le delle Cascine 18, 50144 Florence, Italy; 2 Institute of Bioeconomy (IBE), National Research Council (CNR), Via Gaifami 18, 95126 Catania, Italy; 3 Institute of Bioeconomy (IBE), National Research Council (CNR), Azienda Agraria “Santa Paolina”, S.P. n° 152 Aurelia Vecchia Km 43,300, 58022 Follonica, Italy; 4 Centro Ricerche Metapontum Agrobios-Agenzia Lucana di Sviluppo e di Innovazione in Agricoltura (ALSIA), S.S. Jonica 106, Km 448,2, 75010 Metaponto di Bernalda, Italy; 5 Institute of Bioeconomy (IBE), National Research Council (CNR), Via G. Caproni 8, 50145 Firenze, Italy

**Keywords:** hyperspectral sensors, hyperspectral output optimization, precision agriculture, plant phenotyping

## Abstract

The development of spectral sensors (SSs) capable of retrieving spectral information have opened new opportunities to improve several environmental and agricultural practices, e.g., crop breeding, plant phenotyping, land use monitoring, and crop classification. The SSs are classified as multispectral and hyperspectral (HS) based on the number of the spectral bands resolved and sampled during data acquisition. Large-scale applications of the HS remain limited due to the cost of this type of technology and the technical difficulties in hyperspectral data processing. Low-cost portable hyperspectral cameras (PHCs) have been progressively developed; however, critical aspects associated with data acquisition and processing, such as the presence of spectral discontinuities, signal jumps, and a high level of background noise, were reported. The aim of this work was to analyze and improve the hyperspectral output of a PHC Senop HSC-2 device by developing a general use methodology. Several signal gaps were identified as falls and jumps across the spectral signatures near 513, 650, and 930 nm, while the dark current signal magnitude and variability associated with instrumental noise showed an increasing trend over time. A data correction pipeline was successfully developed and tested, leading to 99% and 74% reductions in radiance signal jumps identified at 650 and 830 nm, respectively, while the impact of noise on the acquired signal was assessed to be in the range of 10% to 15%. The developed methodology can be effectively applied to other low-cost hyperspectral cameras.

## 1. Introduction

Improving the detection and monitoring of ecosystem conditions is a critical issue as the Earth’s biodiversity loss due to human activities is accelerating at an unprecedented rate [1,2]. Recent advantages in data processing and data mining, simultaneously with the development of spectral sensors (SSs) capable of retrieving spectral information from the Earth’s biosphere using the electromagnetic radiation reflected by a terrestrial target, have opened new opportunities in environmental monitoring. Several ecological variables and ecosystem traits have been successfully retrieved from SSs installed on satellite or airborne platforms, e.g., the productivity of forest ecosystems, specific plant traits of crops, and the health status of marine ecosystems [3,4,5]. Advances in precision agriculture techniques have demonstrated how analyzing the spectral information in the electromagnetic spectrum can be a valuable tool to monitor the growth and health status of crops and agricultural products [6]. The SSs have been applied to agricultural production to improve the efficiency of processes during all supply chain operations, such as monitoring leaf water content during crop growth in the field and the quality control process during the post-harvest phases [7,8]. The application of SSs in agriculture field have permitted improvements in several agricultural practices, e.g., crop breeding and phenotyping in high-throughput phenotyping application [9], agricultural land use monitoring and crops classification from satellites or airborne platforms [10,11], cereal yield forecasting [12], and ecosystem services focused on soil and water resources or losses in biodiversity [13]. The SSs are classified as multispectral (MS) and hyperspectral (HS) based on the number of the spectral bands resolved and samples in the same data acquisition, where the simultaneous acquisition of more or less than fourteen spectral bands is considered as the conventional threshold [1,14]. The spectral range suitable for MS and HS instruments is usually classified into several spectral regions, including the visible light spectrum (VIS) characterized by a spectral range from 350 to 700 nm, the near-infrared (NIR) light spectrum ranging from 700 to 1100 nm, and the infrared-shortwave (SWIR) light spectrum highlighted by a light spectrum ranging from 1200 to 2500 nm [14]. SSs are divided in spectrometers and imaging spectrometers. Spectrometers are generally composed of an optical fiber to collect electromagnetic radiation from a defined area, returning a single spectral signature. Imaging spectrometers collect data with a “push broom” line-scan able to compose an image with the movement of the sensor (on aircraft or UAV) or the movement of the target (on industrial conveyor belts) or a “snapshot” approach when an image of n × n pixels is acquired simultaneously [15]. Hyperspectral imaging technologies based on a snapshot sensor consist of an imaging detector that collects an image composed of a specific number of pixels and a defined number of spectral bands. A hyperspectral image (HI) is the result of the acquisition, and each pixel is composed of the spectral information for each band selected [15,16]. The spectral information is expressed in digital counts (DCs), which are also called counts; as a digital unit; or in radiance (mW∙nm^−1^∙sr^−1^∙m^−2^) *as a measure of the intensity of the electromagnetic radiation for a defined area [17]. The data obtained are structured in a 3-dimensional hyperspectral data cube, also described as a hypercube (HC). Here, the image size is the optical resolution of the hyperspectral camera expressed in megapixels (MP) [18], and the spectral resolution is the sampling rate and bandwidth in which the sensor collects the information about the target [19].

HS applications in agricultural activities typically consist of the elaboration of vegetation indices (Vis) obtained by spectral vegetation reflectance, as the the widely used normalized difference vegetation index (NDVI) based on the ratio between red and near-infrared spectral bands [17]. In recent years, several Vis were elaborated and tested to retrieve multiple biochemical parameters, e.g., chlorophyll content, anthocyanins, pigments, and carotenoids, and to monitor biotic and abiotic stress during the crop’s life cycle [20,21]. Therefore, the HS application has progressively become a very interesting tool to acquire data from a broad spatial scale within short intervals of time; remote sensing application are still mostly related to scientific research with some industrial applications, e.g., the production of intelligent farming apps for farmers aimed to provide support for the use of fertilizers, the volume of the irrigation supply, and the characterization of land covers [22,23,24,25]. Nevertheless, the large-scale uses of HS remain limited due to the cost of this type of technology and the technical difficulties in hyperspectral data processing [26].

Since the early 2000s, a new generation of low-cost portable hyperspectral cameras (PHCs) has been progressively developed, increasing the possibilities of deploying applications and developing products given their minor cost, higher versatility, and easiness of application with respect the hyperspectral technologies installed on satellite or airborne platforms and their ability to acquire data with a super-high-spatial resolution (e.g., centimetric or sub-centimetric level) [26,27,28]. Indeed, PHCs can be installed on a ground-based platform or on an Unmanned Aerial Vehicle (UAV) permitting the acquisition of hyperspectral data at narrow temporal acquisition intervals on a defined area [28,29]. The low-cost devices reported the following characteristics. PHCs are composed of a frame detector to acquire spectral images into a specific field of view (FOV) expressed in degrees, which defines the spatial resolution of the instrument [27,30]. The spectral resolution is calculated by the full width at half maximum (FWHM) of the spectral sensor, where FWHM is defined as the width of a spectral band below which the signal would overlap [31]. Imaging chip technology commonly found in the PHCs are charged coupled devices (CCD) and complementary metal oxide semiconductors (CMOS). Both sensor types allow the acquisition of a million pixels in a single image with high spectral and spatial resolution. Nevertheless, CMOS sensors show higher sensitivity and low power consumption but also higher background noise and dark current [32]. The PHCs collect images using the natural light, and the imaging system cannot function in a dark or low-light environment [33]. Despite the technological developments of the low-cost PHCs, critical aspects are reported during acquisition and data processing, such as the long instrumental warm-up time, the presence of spectral discontinuities in correspondence of the spectral border region of different CMOS sensors, signal jumps, and high levels of background noise. The unavoidable limitations due to the low cost cannot be resolved with technology; thus, it is crucial to reduce them with careful and optimized data processing and sensor operation [34,35,36].

In this work, the low-cost PHC Senop HSC-2 (HSC-2) and the Ocean Optics USB 2000 reference spectrometer with a VIS/NIR spectral range were used to collect hyperspectral data on a white reference and a vegetation target during multiple acquisitions conducted under controlled conditions during a phenotyping experiment. The spectral outputs obtained using the PHC and the spectrometer were used to develop a novel data processing pipeline capable of improving the PHC data quality and calibrated spectral radiances. Subsequently, critical issues associated with the PHC, such as spectral signal jumps, dark current magnitude and temporal variability, dark current noise level and noise impact on the radiometric signal, and measurement limits due to the loss of sensitivity by the CMOS sensors, were analyzed and discussed. The correction methodology proposed here is suitable for use on other types of PHCs affected by similar issues and for applications other than plant phenotyping.

## 2. Materials and Methods

### 2.1. Experimental Activities

The work activities were conducted during the years 2020 and 2021 at CNR (National Research Council of Italy) in the facilities of Florence and Follonica (Italy) as well as ALSIA (Agenzia Lucana di Sviluppo ed Innovazione in Agricoltura, S.S. Jonica Km 448,2, 75012 Metaponto MT, Italy), a node of the European Plant Phenotyping Network (EPPN) (https://emphasis.plant-phenotyping.eu, accessed on 20 June 2023).The experimental activities, consisting of the simultaneous hyperspectral acquisitions from a reference and vegetation targets, were conducted using the HSC-2 and the USB 2000 reference spectrometer.

### 2.2. Hyperspectral Devices

#### 2.2.1. Ocean Optics USB 2000 Reference Spectrometer

**Figure 1:**
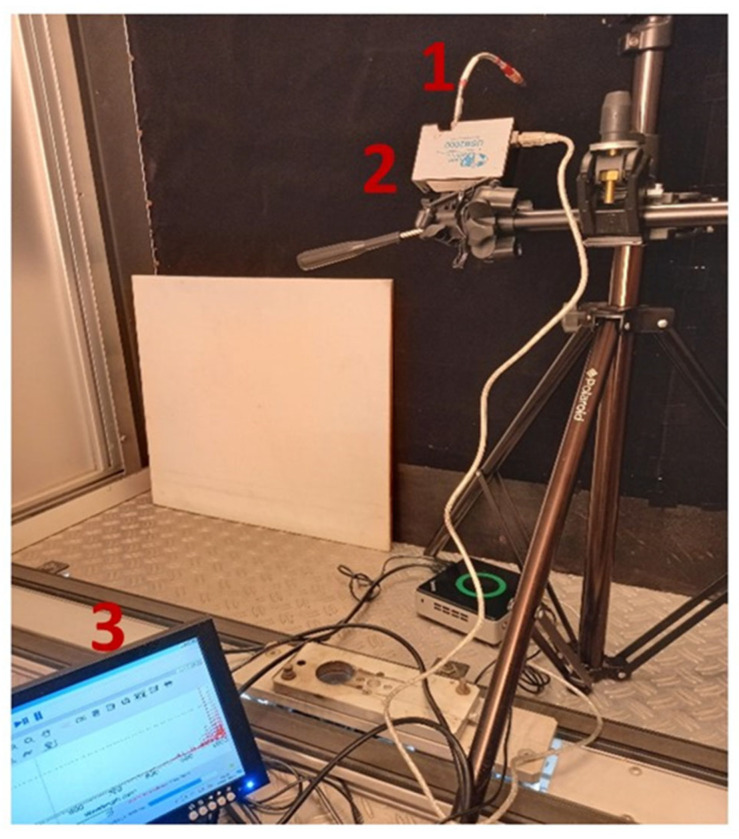
USB 2000 acquisition set. The electromagnetic radiations are collected using an optical fiber (1) connected to the body of the spectrometer (2). The hyperspectral data can be visualized online using the Ocean View software version 2.0.7 (3) (Ocean Insight, Rochester, NY, USA).

The USB 2000 (Ocean Insight, Rochester, NY, USA) is a portable spectrometer able to collect continuous spectral information from a surface (Figure 1). The USB 2000 is characterized by a spectral sampling interval ranging from 350 to 1000 nm with 0.2 nm of spectral resolution. The spectrometer is based on a Sony ILX511 linear silicon CCD array (Ocean Insight, Rochester, NY, USA).

#### 2.2.2. Hyperspectral Camera Senop HSC-2

**Figure 2:**
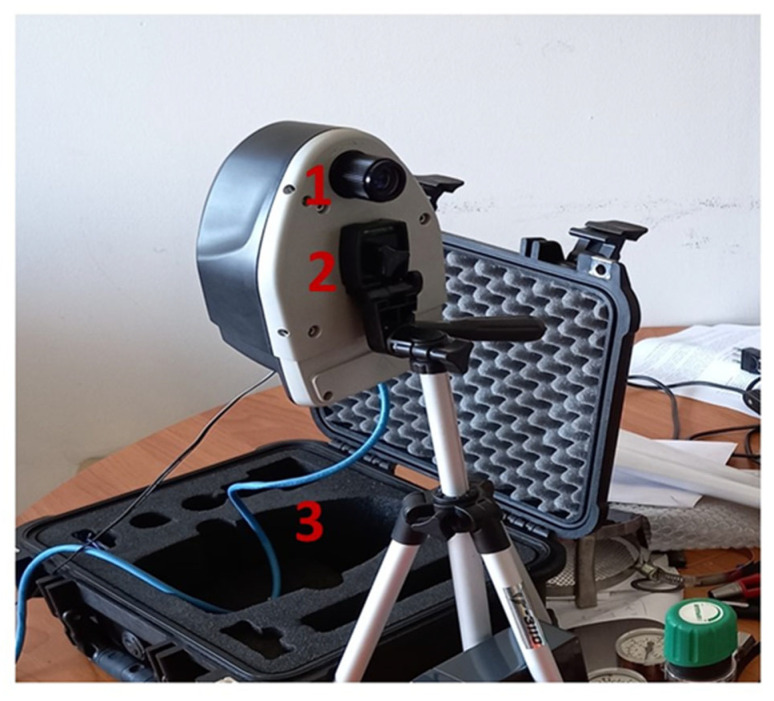
Hyperspectral camera HSC-2. The HSC-2 is a frame-based digital hyperspectral camera equipped with a true global snapshot sensor that can collect the entire 3-dimensional data cube in a single integration period (1). The operator can set several acquisition parameters, such as spectral range and resolution, and exposure. The hyperspectral device has a total weight of 990 g, and it can be installed on a tripod (2). The hyperspectral data, combined in a HC, are stored in an internal memory of one terabyte size. The instrument can transfer the hyperspectral data through an ethernet connection (3).

The HSC-2 (Senop Oy, Kangasala, Finland) (Figure 2) is a hyperspectral device with snapshot approach and a VIS/NIR spectral range already used in research activities, e.g., the acquisition of high-resolution hyperspectral images acquired in flight by a UAV platform [29,37], the assessments of plant health status through the detection of potassium concentration on leaves [38], and the development of a pathological tissue database in biomedical applications [39,40].

The hyperspectral data cube can be explored and converted in actual radiance (mW∙nm^−1^∙sr^−1^∙m^−2^) by means of gain values specific for each spectral band adopted using the software Senop (version HSI-2 2019.04.10.1) HIS-2 provided by the manufacturer. At the end of every acquisition, the derived HC was stored in a specific directory containing the raw HC and the metadata necessary to handle the spectral information contained in the HC, such as the gain values applied to convert the HC saved in DCs in the radiometric units, the FWHM for each band, and the sampled measurement bands. The general specification for optics, image size, and spectral capability of the instrument are reported in Table 1.

### 2.3. Hyperspectral Correction Methodology

**Figure 3:**
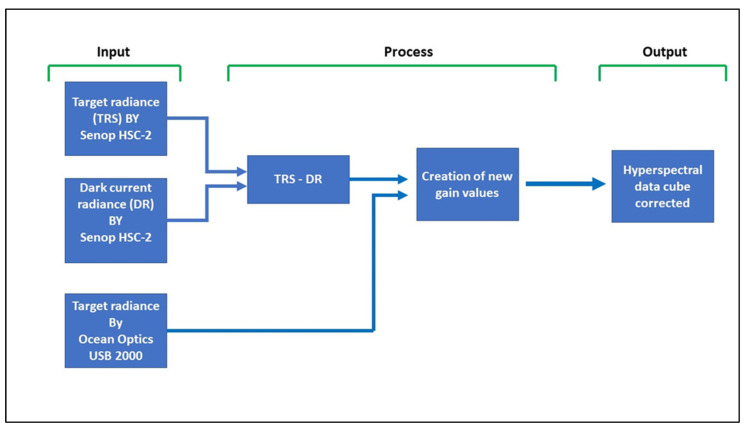
Workflow of the correction methodology.

The hyperspectral correction methodology was specially designed and developed (i) to take into account the background dark current signal generated by the HSC-2 and (ii) to calculate the corrected correction factors (CF) to be applied to DCs obtained using the HSC-2 and so resulting in corrected radiance values. The correction methodology was developed using Matlab R2021a (MathWorks, Natick, USA), and the related algorithm is called “ImportSenop”. The workflow of the correction methodology is reported in Figure 3.

#### 2.3.1. Inputs

The methodology requires three inputs: (1) The target signal in DC obtained with the reference target using the hyperspectral camera HSC-2; (2) The dark current signal in DC obtained using the HSC-2 in complete darkness conditions, which is obtained by covering the HSC-2 optic; (3) The calibrated radiance obtained with the reference target using the USB 2000. The spectral output of the USB 2000 was interpolated at the exact spectral bands of the HSC-2 to consistent and comparable spectral signatures.

#### 2.3.2. Process

The first operation of the pipeline is an automatic subtraction of the DC dark current values from the DC target signal values (Figure 3) for each pixel and for each spectral band. 

Subsequently, for each spectral band, the radiance value obtained using the USB 2000 was divided by the corresponding DC value measured by the HSC-2 to obtain the CF, which is to be used in place of the original gain value reported in the header of the metadata file generated for each HSC-2 snapshot. This operation is based on the MATLAB function interp1. Subsequently, a 3-dimension signal filter working both in the spectral and spatial domains was applied to the HC using the medfilt3 MATLAB function. 

#### 2.3.3. Outputs

The result of the correction methodology is a set of CF values strictly related to the set of adopted spectral bands and to the camera setup in terms of optic aperture and exposition time. The output of the pipeline is a new hypercube composed of the corrected radiance value for each spectral band. In addition, the function can be set to convert automatically the radiance obtained in reflectance (Ref) as follows:

Ref = Rad/Rw(1)

where Rad is the radiance obtained using the target, and Rw is the radiance obtained using the white reference target.

### 2.4. Hyperspectral Correction Methodology Test

#### 2.4.1. Acquisition Setup

**Figure 4:**
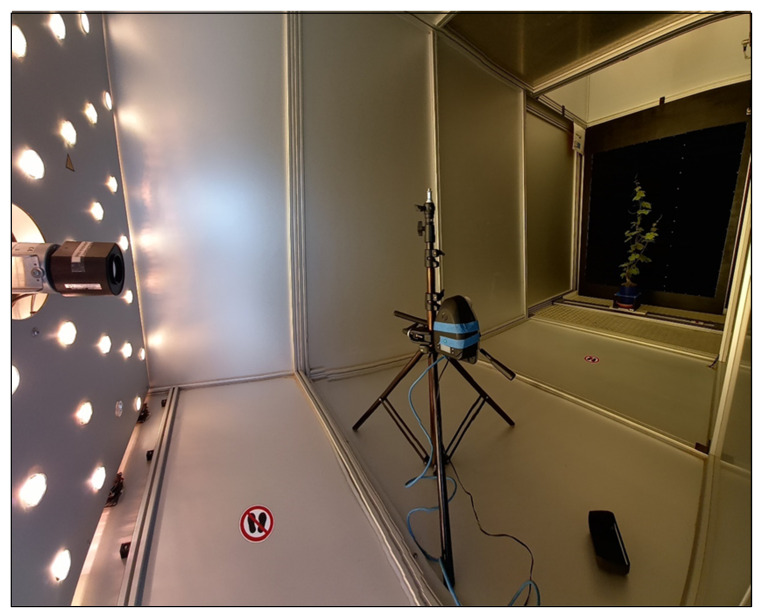
The acquisition chamber used in the experiment. In the image, the light source, the HSC-2, and a vegetation target are visible in the background.

The efficiency of the correction methodology developed here was computed during an acquisition test. The acquisition setup used during the test was composed of an acquisition chamber (Figure 4) based on the LemnaTec Scanalzer 3D system (LemnaTec GmbH, Aachen, Germany) [41]. A white Lambertian reference target with a 75% reflectance was placed in the acquisition chamber in an orthogonal position compared to the light source. The light source comprised 120 Osram GmbH halogen lamps with a maximum level variation of 2%. Every lamp produced a net power of 35 Watts. The USB 2000 was placed a few inches from the reference at an angle of 45° to the reference target. During the acquisition process, the HSC-2 was installed with a horizontal camera axis at 1.50 m from the white reference target in a nadiral position with respect the target and light source. The exposure was set to 80 milliseconds for every spectral band. The HSC-2 underwent a warm-up time of 20 min to stabilize thermal operating conditions.

#### 2.4.2. Dark Current Computation and Analysis

The HSC-2 instrumental background signal, also called dark current, was assessed through multiple acquisitions conducted every 10 min for a total of 130 min, posing the optic of the hyperspectral camera in complete darkness condition. After every dark current acquisition, the optics dark enclosure was removed, and the white reference target was immediately acquired. The dark current and reference signals were processed as the mean value of the HSC-2 full spectrum, expressed in DC units. Finally, the dark current and reference trends were compared to retrieve the impact of the dark current signal on the total radiance in different conditions.

#### 2.4.3. Calibration on a White Reference Target

The hyperspectral correction methodology was calibrated using the white reference target previously described. The reference target was acquired using the HSC-2 and the USB 2000 spectrometer. Subsequently, the trend of the radiance obtained using the USB 2000 was compared to the HSC-2 radiance computed using the radiance correction methodology developed here.

#### 2.4.4. Dark Current and White Reference Noise Assessment

The radiometric signal noise level was estimated for the dark current and the white reference target, representing the two extremes of null and full reflectivity, respectively. The noise for selected spectral bands was computed as the variance of the frequency distribution of the camera output in DC units, after subtracting the average. A Gaussian distribution was fitted on the noise frequency distribution to assess whether it well represents experimental data. The contribution to total noise measured on the white reference target was partitioned into a component related to the dark current and a component related to the radiometric signal itself, under the assumption of Gaussian noise distribution as follows:

σ_WHITE_^2^ = σ_DARK_^2^ + σ_RAD_^2^ (2)

where σ_WHITE_^2^ and σ_DARK_^2^ are the variances measured on the white reference and dark current signals, respectively, and σ_RAD_^2^ is the variance of the radiometric signal computed using the Equation (2).

#### 2.4.5. Test on a Vegetation Target

The proposed correction methodology was finally tested on a vegetation target. A maize plant was positioned in the acquisition chamber using the acquisition setup previously described. The hyperspectral camera was set to its maximum spectral range from 400 to 1000 nm for a total of 203 spectral bands.

The acquired hyperspectral dataset was post-processed to obtain three outputs:

- The hypercube computed in DC units.
- The HC converted in radiance units, using the original gain values reported in the HSC-2 header file.
- The HC computed in radiance units obtained using the novel CF after dark current correction.

Finally, the results obtained using the three different processes were compared and discussed.

## 3. Results and Discussions

### 3.1. Dark Current Assessment

**Figure 5:**
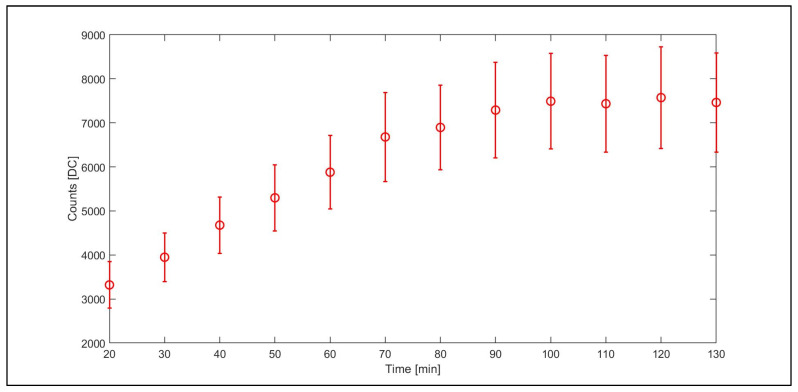
Mean of the dark current values expressed in DC (counts) units for a sample area (100 × 100 pixels) for a total of 203 spectral bands from 400 to 1000 nm. The values were obtained from a time ranging from 20 to 130 min. The first 20 min were dedicated to the instrument warm-up, and the data were not reported. The standard deviations of the spectral data cube are reported as error bars.

The dark current average value (Figure 5) constantly increases for the first 90 min of the acquisition, showing a stabilization at a value that is approximately double the initial dark current value. Indeed, at the start of the experiment a mean dark value near 3000 digital counts was registered. At the end of the experiment, a dark value near 7000 digital counts was registered highlighting the presence of a relatively long warm-up phase. Variable warm-up times were reported for other hyperspectral cameras, e.g., Zhu et al. [35] reported 30 min as sufficient warm-up time for the “GaiaSorter” I system produced by Zolix Co., Ltd. (Beijing, China). The dark current appears stable from 90 min to the end of the experiment.

**Figure 6:**
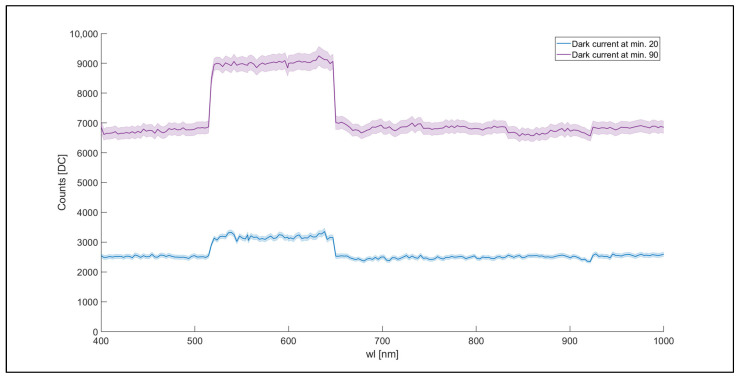
Example of the dark current HSC-2 spectral signatures obtained at 20 and 130 min. Each spectral signature (reported in DC) for each spectral band is the mean of data from 1024 × 1024 pixels (HSC-2 full spatial resolution). The standard deviation of the image acquired in each spectral band is reported as shaded area.

The spectral signature of the dark current was computed, and the results registered at 20 and 130 min from the beginning of the experiment are reported in Figure 6.

**Figure 7:**
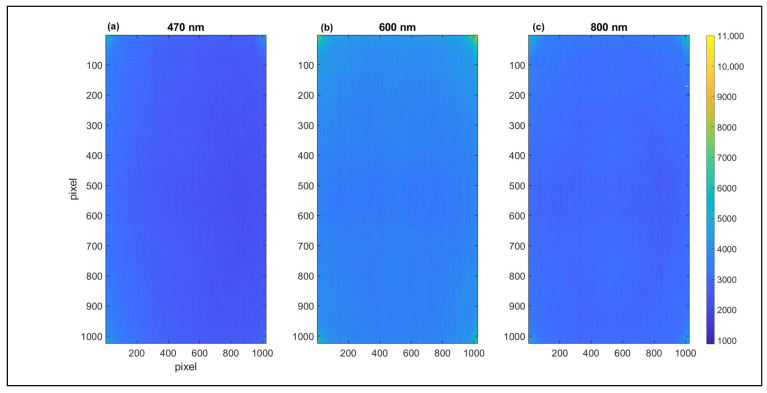
HIs obtained using a dark current acquisition collected after 20 min from the start of the experiment. The HIs were acquired from the three spectral regions at 470 (**a**), 600 (**b**), and 800 nm (**c**). The HIs were obtained with 1024 × 1024 pixel size (HSC-2 full spatial resolution), and each pixel shows a DC level evidenced by the color bar.

The spectral signature shows a pattern clustered on three spectral regions (from 400 to 513 nm, from 513 to 650 nm, and from 650 to 1000 nm, separately) divided by two spectral jumps at 513 and 650 nm. The three regions and the associated signal jumps are likely related to the presence of two spectral detectors that the camera contains. The spectral regions from 400 to 513 nm and 650 to 1000 nm show a mean of 2000 and more than threefold (6900 digital counts) for the 20 and 130 min measurements, respectively. In both the intervals, the measured standard deviation was higher at 130 min than at 20 min, and the related coefficient of variations (standard deviation/mean value) were 12.5% and 7.2%, respectively. A similar behavior was observed within the spectral region from 513 to 650 nm with a mean value near 3100 and 9300 digital counts at 20 and 130 min, respectively. Again, the related coefficients of variation were 12.5% and 7.2%, respectively. To assess the possible presence of dark current spatial patterns on the detector, the HI obtained from the three spectral regions defined above is reported in Figure 7.

Results show differences between HIs regarding sharpness, uniformity, and background noise level. Subfigure b shows lower DC values and lower uniformity of the pixel’s DC values than subfigures a and c in agreement with results obtained in Figure 6. Figure 7b shows higher levels of background noise than Figure 7a,c reporting image discontinuities highlighted by vertical stripes. The HIs obtained at 470 and 800 nm (Figure 7a,c) show higher image sharpness and uniformity compared to the HI at 600 nm. The HI at 470 nm (Figure 7a) shows a decrease in the DC level from the left to the right side of the image (from light blue to dark blue). Generally, the HI at 800 nm appears more uniform than the HIs at 470 and 600 nm. The lower image uniformity in the edge spectral regions and the spectral jumps at 513 and 650 nm could be interpreted as an effect of the presence of different CMOS sensors, affecting the acquisition of sharpness HIs in the edge spectral regions [33].

**Figure 8:**
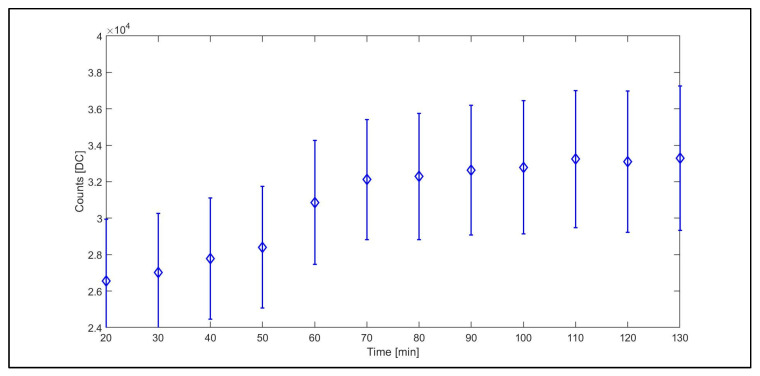
Mean of the white reference target expressed in DC (counts) units for a region of interest (ROI) of 100 × 100 pixels in size for a total of 203 spectral bands from 400 to 1000 nm. The ROI was the same as that noted in Figure 5. The standard deviation is reported as error bars.

A white reference target was acquired simultaneously with the dark current acquisitions, and results are shown in Figure 8.

The DC values obtained using a white reference target constantly increased for the first 90 min of the acquisition, according to the results obtained in dark condition. Indeed, from 20 to 90 min, a progressive increase in the magnitude of the DC values was reported. The similar trend between Figure 5 and Figure 8 is due to the effect of the dark current signal on the total signal; therefore, considering the dark current trend and its temporal dynamic during the warm-up phase is essential to obtain a clean spectral signal [34,35]. Our results reveal an increasing dark current signal with time during the warm-up phase following the camera switch on, until a stabilization is reached after about 90 min. Although specific to the camera model object of this study, this pattern is likely characteristic of similar sensors that do not implement a robust detector cooling system or a thermal stabilization hardware. In terms of operational guidelines, on the one hand, using the camera in the initial phase when its temperature is still relatively low would result in a lower dark current signal. On the other hand, this condition would imply dealing with a dark current signal that is highly unstable and increasing over time. Performing the acquisition after thermal stability is reached would instead result in a large but stable dark current signal to be managed. Moreover, our results showed how the dark current changes its value for each pixel and wavelength and must be processed on a pixel-by-pixel basis. Therefore, dark current acquisition and subtraction from the target image is always recommended. However, for warm-up times less than 90 min, it must be frequently repeated, considering the rapid changes in the dark current values associated with the increasing temperature.

#### Noise Assessment

**Figure 9:**
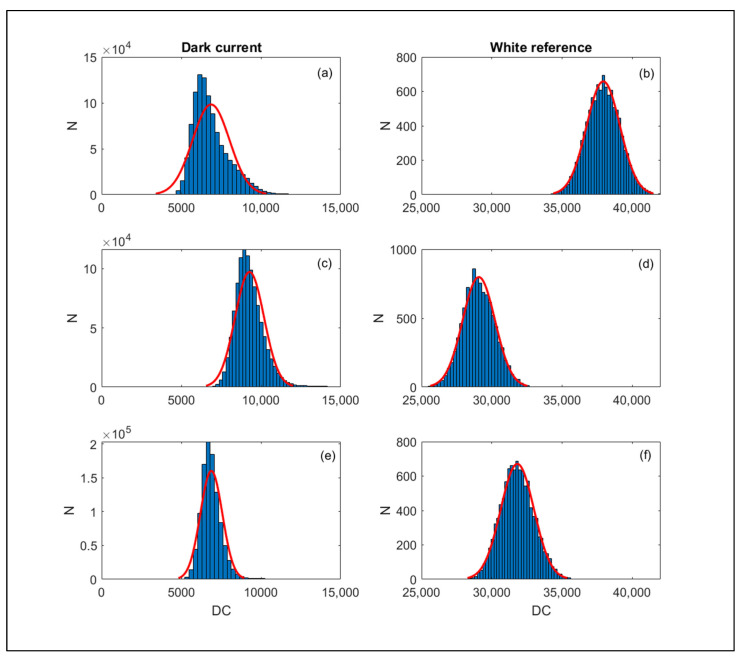
DC frequency distributions of the dark current ((**a**,**c**,**e**) panels, entire data image) and white reference (**b**,**d**,**f**) panels, 100 × 100 region corresponding to the reflective target) at the three spectral bands: 470 nm (**a**,**b**), 600 nm (**c**,**d**), and 800 nm (**e**,**f**). A Gaussian law was fitted to each distribution (red curve).

The noise level of the dark current signal and the white reference signal, computed at the same three selected wavelengths as noted in Figure 6 and Figure 7, was reported in Figure 9 as frequency distribution of the DC counts. The figure shows that overall the distributions are well represented by a Gaussian law at all wavelengths for the white reference and at 600 and 800 nm for the dark current; however, it is skewed toward the right-end tail at a wavelength of 400 nm (Figure 9a). The assumption of a Gaussian distribution could overall be adopted to estimate noise level as the variance of the distribution and compute the noise contribution of the dark signal on the total acquired signal using Equation (2). During the warm-up, the variance of the dark current signal, representing its noise level, increased with the same pattern as the signal itself, representing about 15% of the signal (Figure 5). Over the white reference target, such a level decreased to about 10% of the signal (Figure 8). By inverting the error propagation from the dark and white measurements under the assumption of a Gaussian noise distribution using Equation (2), we estimated that the noise σ_WHITE_^2^ affecting the white reference signal after the stabilization is reached (e.g., 120 min) with 10.7% originating from the dark current noise σ_DARK_^2^ and the remaining 89.3% from the radiometric signal noise σ_RAD_^2^, representing the noise associated with the radiometric signal conversion and processing. On intermediate reflective targets that are commonly encountered, we can therefore expect a total noise impact on the raw DC signal within this range of 10% to 15%, stable across spectral bands as highlighted in Figure 6.

### 3.2. Hyperspectral Correction Methodology Application

#### 3.2.1. Application of the Hyperspectral Correction Methodology on a White Reference Target

**Figure 10:**
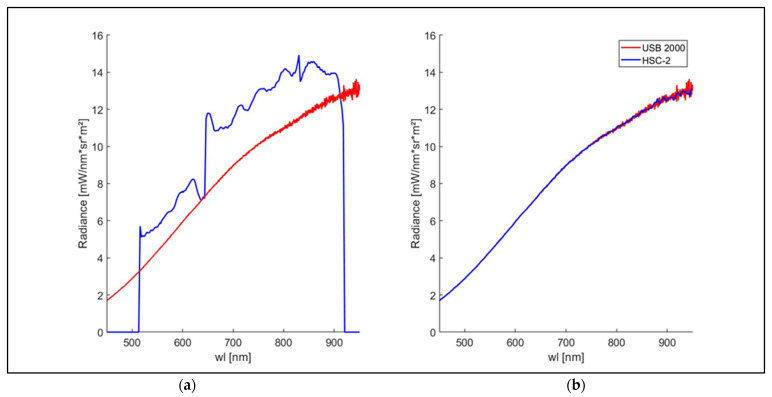
USB 2000 and HSC-2 spectral radiances (mW∙nm^−1^∙sr^−1^∙m^−2^) obtained with the standard gain values for HSC-2 (**a**) and with gain values obtained using the CF procedure (**b**). The spectral data were obtained in a spectral range from 450 to 950 nm.

The light reflected by the white reference target was acquired using the HSC-2 and the USB 2000 spectroradiometer. The respective radiances were computed both using the original gain values and the new gain values obtained using the CF procedure described below (Figure 10).

The USB 2000 and the HSC-2 radiances before the application of the CF showed differences in trend and magnitude. The light spectrum obtained using the USB 2000 appears linear and homogeneous, reproducing a halogen lamp’s characteristic light spectrum, which shows a steady increase from VIS to NIR [42]. Instead, the radiance obtained using the HSC-2 shows several magnitudes jumps at the same wavelengths where jumps in the dark current signal were also detected. In the spectral region from 450 to 515 nm, the HSC-2 radiance equals zero, which is in the spectral region from 920 to 950 nm (plot a, blue radiance). This result is due to the gain values automatically generated by HSC-2, which are equal to zero in the same spectral regions. Two radiance jumps are shown around 650 nm and 830 nm. 

After applying the correction methodology, the HSC-2 radiance matches the USB 2000 radiance as a result of the calibration procedure (Figure 10b).

#### 3.2.2. Application of the Hyperspectral Correction Methodology on a Vegetation Target

**Figure 11:**
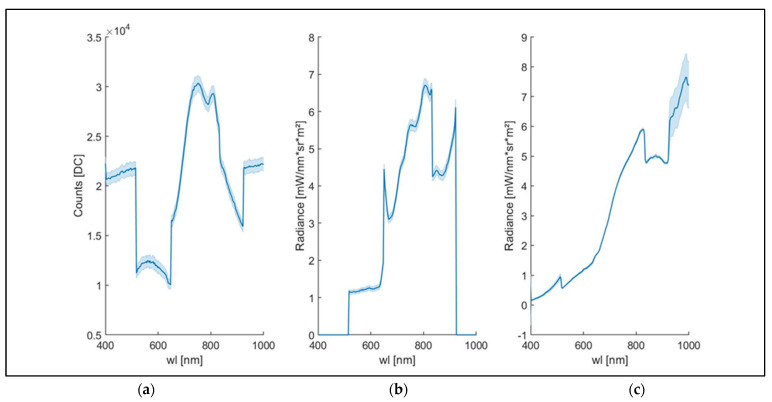
Three spectral signatures obtained using the same ROI (11 × 11 pixels, for a total of 121 pixels) chosen from a leaf in a nadiral position compared to the light source and the optic of the HSC-2. The spectral signatures were reported as follows: (1) As DC (plot (**a**)) automatically generated by HSC-2. (2) As radiance (mW∙nm^−1^∙sr^−1^∙m^−2^) obtained using the gain values in the HSC-2 header file (plot (**b**)). (3) As radiance (mW∙nm^−1^∙sr^−1^∙m^−2^) obtained applying the novel CF (plot (**c**)). The spectral data were acquired from 400 to 1000 nm, using the full spectral range of the HSC-2. Standard deviation is reported.

The correction methodology was tested on a vegetation target. Results are shown in Figure 11.

The spectral results in DC units (plot a) showed signal stability in the spectral range from 400 to 513 nm and above 930 nm, where DC values near 2.2 × 10^4^ were reported. The DC trend (Figure 11a) showed a signal negative jump of approximately 1.0 × 10^4^ DC at 513 nm and a positive jump of approximately 0.6 × 10^4^ DC at 630 nm. Signal jumps were observed at 513, 650, 830, and 930 nm of approximately 1.0 × 10^4^, 0.6 × 10^4^, 0.4 × 10^4^, and 0.6 × 10^4^ DC, respectively. These jumps are likely related to different CMOS sensors in the VIS and NIR spectral range, highlighting one of the main limits of the low-cost PHC based on CMOS technology [33,34,43]. 

The radiance results obtained using the gain value provided by the HSC-2 header file are shown in plot b. The radiance values are equal to zero from 400 to 513 nm, and this result is due to the gain values automatically generated by HSC-2, which are equal to zero from 400 to 513 nm and 920 to 1000 nm, according to the results described in the previous paragraph. The radiance results show a plateau in the spectral range from 513 to 650 nm (plot b) before the slope increase in the red-edge spectral region, as described in plot a. Generally, the spectral discontinuities highlighted in plot a are not reduced by radiance conversion obtained using the gain values automatic generated in the header file.

The spectral results obtained by applying the novel CF are shown in plot c. The shape of the spectral signal after the application of the CF showed an increase in slope in the spectral region from 650 to 820 nm, in accordance with the well-known slope increase in the red-edge spectral region on vegetation due to photochemical processes related to chlorophyll light absorption [3,44]. The application of the CF resulted in nonzero radiance values in the two spectral ranges from 400 to 513 and 920 to 1000 nm. The radiance trend obtained by the application of the CF, compared to plot b, showed a peak near 500 nm that corresponded with the pigment’s absorption spectral regions, in accordance with the spectral leaf pigments analysis results (e.g., chlorophylls, carotenoids, and anthocyanins) reported in the literature [45,46,47]. Nevertheless, the peak highlighted appears weak and not very sharp, probably showing a measurement limit due to the CMOS VIS sensor previously hidden by the gain value in the HSC-2 header file (imposed equal to zero). The application of the CF allowed a clear spectral signal to be obtained due to a noise reduction compared to the results shown in plot b. Indeed, the spectral signal has reported lower fluctuations and more stability than the results obtained using the gain value generated by the header file. Generally, the standard deviation in plot c is lower than that noted in plots a and plot b, and the application of the CF has reported a reduction of the noise effects in the spectral signature compared to the results obtained using the gain values automatically generated in the header file. Finally, the CF proposed here has significatively reduced the radiance jumps due to the operation limits of the two CMOS sensors near 650 nm and 820 nm, improving the precision and reliability of the low-cost PHC tested here. Specifically, while the spectral jumps at 513 and 930 nm could not be compared given that the factory calibration did not provide spectral data before and after those bands (Figure 11b), the jumps at 650 and 830 nm were reduced by factors of 99% and 74%, respectively (Figure 11c).

## 4. Conclusions

This work presents a novel methodology to improve the performance of a low-cost PHC. The performances of the PHC HSC-2 were explored and tested through hyperspectral acquisitions conducted on a white reference and a vegetation target under controlled conditions of light and an acquisition set. The spectral results obtained using the HSC-2 were compared with the spectral results obtained using the USB 2000. Subsequently, a novel hyperspectral correction methodology was successfully developed and tested. Therefore, the conclusions of this study are as follows:

- A low-cost PHC can be a powerful tool in hyperspectral applications. However, the poor sensitivity of the two CMOS sensors in the margin’s sides of the VIS/NIR spectral regions from 400 to 513 and 920 to 1000 nm contributed to reducing the performance of the PHC. In addition, several signal gaps were identified as falls and jumps across the spectral signatures near 513, 650, and 930 nm.
- The dark current signal magnitude increases over time. Therefore, the instrument’s warm-up time must be considered, and applying an image correction based on frequent dark current acquisitions is strongly recommended to obtain a clean spectral signal.
- The hyperspectral correction methodology developed in this work significantly improves the qualities of the spectral output obtained using the PHC HSC-2. Indeed, the radiance jumps and the signal noise were reduced, especially in the spectral region from 650 to 830 nm.

This work can stimulate further studies to assess the proposed correction methodology under different acquisition conditions and to investigate its sensitivity to parameter settings and environmental conditions [48].

A framework for a sensitivity analysis to be performed on this and similar cameras should be composed of the following:

- Temperature static sensitivity: assess the variations of the method outputs (e.g., the optimized set of band specific correction factors [CFs]) under a range of ambient temperature conditions in a controlled environment, allowing the camera to stabilize under each temperature condition;
- Temperature dynamic sensitivity: assess the variations in CFs under temperature variations in a controlled environment, as temperature ramps that are likely to be encountered in real applications;
- Stability: assess the variations in CFs by operating the camera in the same controlled conditions at different times;
- Parameter sensitivity: assess the variations in calibrated spectral radiances related to the variations in CFs obtained from the previous steps;
- These steps would permit estimations of the band-specific uncertainty of spectral radiances and assessments of the temporal frequency at which updated CFs should be computed. Additional studies are also recommended to test the methodology on various spatial scales through field experiments conducted using fixed platforms or UAVs and on different natural and artificial targets spanning from low to high reflectivity. The results of this work can be applied to other areas of interest, such as soil composition retrieval, material property detection, and biomedical applications, and other low-cost hyperspectral cameras that may suffer from the same limitations.

## Figures and Tables

**Table 1:** Specifications of the HSC-2.

Optics	Imaging Capability	Spectral Capability
FOV 36.8 degrees. Focus distance: 30 cm to ∞, limited FOV with less than 30 cm distances.	Image frame size: 1024 × 1024 pixels.	Wavelength area: from 400 to 1000 nm.

## Data Availability

The data presented in this study are available on request from the corresponding author.

## Abbreviations

**Abbreviation**	**Description**
CCD	Charged coupled devices
CF	Correction factors
CMOS	Complementary metal oxide semiconductors
DC	Digital counts
FOV	Field of view
FWHM	Full width at half maximum
HC	Hypercube
HI	Hyperspectral image
HSC-2	Senop HSC-2
MP	Megapixel
MS	Multispectral
NDVI	Normalized difference vegetation index
NIR	Near-infrared
PHCs	Portable hyperspectral cameras
SSs	Spectral sensors
SWIR	Shortwave infrared
UAV	Unmanned aerial vehicle
VIS	Visible light spectrum
VI	Vegetation index
